# Feasibility and user experience of the unguided web-based self-help app ‘MyDiaMate’ aimed to prevent and reduce psychological distress and fatigue in adults with diabetes

**DOI:** 10.1016/j.invent.2021.100414

**Published:** 2021-06-15

**Authors:** Linda T. Muijs, Maartje de Wit, Hans Knoop, Frank J. Snoek

**Affiliations:** a Department of Medical Psychology, Amsterdam Public Health research institute, Amsterdam UMC, Vrije Universiteit Amsterdam, the Netherlands; b Department of Medical Psychology, Amsterdam Public Health Research Institute, Amsterdam UMC, University of Amsterdam, the Netherlands

**Keywords:** Psychological distress, Fatigue, Diabetes mellitus, Web-based app, Cognitive behavioral therapy, Self-help

## Abstract

**Introduction:**

Psychological distress and fatigue are common in persons with diabetes, adversely affecting quality of life and complicating diabetes self-management. Offering diabetes-specific self-guided cognitive behavioral therapy (CBT) may be helpful for persons with diabetes and mild symptoms of psychological distress and fatigue. We are the first to test the feasibility and user experiences of a web-based self-help app called ‘MyDiaMate’ in adults with type 1 and type 2 diabetes.

**Methods and materials:**

MyDiaMate was developed in close collaboration with persons with diabetes and professionals, building on elements from existing (guided) diabetes-specific CBT interventions. The study was advertised, offering free access to the app for adults with diabetes for a period of three months. Feasibility and user experiences were tested in a non-randomized study with pre- and post- measurements and interviews in a small sample.. In addition usage of the app was studied using log-data..

**Results:**

In total *N* = 55 adults with diabetes signed up for the study. Mean age was M = 42.7 (SD = 15.6), mostly women (*n* = 39, 70.9%), higher educated (*n* = 36, 65.5%), and diagnosed with type 1 diabetes (*n* = 37, 67.3%). About half reported current or a history of psychological complaints. All the participants completed baseline assessments, and *n* = 32 participants (58%) completed the follow-up questionnaire. Main reasons for participating in the study were: to preserve or improve mental fitness (40.6%), curiosity (25.0%) and wanting to contribute to research (34.4%). No major technical issues were encountered in accessing or using the app. The app was opened at least once by *n* = 51 participants, median use of the modules was 28 min (1–80) within a period of 1 to 92 days (median = 10). Almost all participants (*n* = 50, 98.0%) opened the basic module ‘Diabetes in balance’, of whom 32 (62.7%) completed this module. ‘My mood’ and ‘My energy’ were opened by *n* = 40 (78.4%) and *n* = 32 (62.7%) participants, respectively, and completed by *n* = 21 (52.5%) and *n* = 9 (28.1%) of the participants. Of all participants, 40.6% would recommend the app to others living with diabetes.

**Conclusions:**

This study confirmed the feasibility of MyDiaMate as a diabetes-specific self-guided app for adults wishing to preserve or improve their psychological health. While user experiences were overall positive, further tailoring the content to individual needs and preferences could enhance uptake, usage and appreciation. Future research should explore its effectiveness in a randomized controlled trial.

## Highlights

• MyDiaMate is the first diabetes-specific self-help app to help prevent and reduce psychological distress and fatigue.
• The study shows promising results in terms of feasibility and user experiences of the app.
• Personalizing content may further improve engagement and appreciation.
• Further research should test the efficacy with regard to reduction and prevention of psychological distress and fatigue.

## 1 Introduction

Self-management is the cornerstone of overall diabetes management, aiming for near normal blood glucose levels in order to reduce the risk of acute blood glucose excursions and disabling long-term complications (Young-Hyman et al., 2016). The continuous need to self-regulate blood glucose in the face of the social, behavioral, and emotional challenges of daily life is psychologically burdensome and can lead to ‘diabetes burnout’ (Polonsky, 1999). Diabetes-related distress (diabetes distress) is highly prevalent in people living with diabetes and associated with adverse health outcomes (Perrin et al., 2017; KMP et al., 2010; Fisher et al., 2010). Moreover, depression, anxiety and chronic fatigue are common among both persons with type 1 and type 2 diabetes (Smith et al., 2013; Roy and Lloyd, 2012; Fisher et al., 2012; Pouwer et al., 2005; Menting et al., 2016; Park et al., 2015; Goedendorp et al., 2014). However, despite increasing evidence of effective psychological therapies, access to professional psychological support as part of diabetes care is limited in many countries (Young-Hyman et al., 2016; Peyrot et al., 2013). This ‘gap’ could potentially - at least partly- be bridged by offering effective self-help interventions tailored to the psychological needs of people with diabetes (Snoek, 2020).

Indeed, Internet-based and Mobile based interventions (IMI) allow for broad dissemination of psychological support for persons with diabetes at relatively low costs (Donker et al., 2015; Baumeister et al., 2014). Particularly for prevention purposes and reduction of mild psychological problems, unguided internet-interventions may be appropriate and preferred, given its low intensity, flexibility (usage is not constrained by time and place), anonymity and privacy (e.g. less perceived stigmatization) and allow for scalability (Franco et al., 2018). Specifically, mobile applications (apps) offer the opportunity to provide remote support, which is especially relevant and suitable for persons with diabetes who are continuously confronted with psychological challenges (Snoek, 2020; Baptista et al., 2019). Yet, for people with diabetes, almost all the available apps provide instrumental support, aimed at lifestyle changes and improving glycemic control (Kitsiou et al., 2017) but do not tap into behavioral and psychosocial issues, e.g. adaptive coping, problem solving strategies, known to be of crucial importance for adequate diabetes self-management (Ye et al., 2018).

Efficacious cognitive behavioral therapy (CBT) interventions have been developed for persons with diabetes experiencing diabetes distress, some of which are internet-based (Schmidt et al., 2018). Guided internet CBT-based interventions for depression and chronic fatigue have shown to be effective in persons with diabetes with the opportunity to ‘blend’ with face-to-face sessions (van Bastelaar et al., 2011; Ebert et al., 2017; Menting et al., 2017). Interestingly, guided self-help programs based on CBT for persons with diabetes and depression proved effective also in the more severe clinical cases (van Bastelaar et al., 2012; Schlicker et al., 2019). However, for a large group of persons with diabetes reporting mild to moderate levels of psychological distress or fatigue, low intensity CBT programs could suffice, characterized by a minimum level of intervention time and no health care professional support, to create maximum gain (Bennett-Levy et al., 2010).

Whether web-based self-help for persons with diabetes with mild psychological distress or fatigue is feasible and effective, has not been researched yet. This lead us to develop an unguided self-help app, called ‘MyDiaMate’, for adults with type 1 and type 2 diabetes to assist help prevent and reduce psychological distress and fatigue. Here we report on the development of the app and a feasibility study.

The objectives of the study were 1) to test feasibility of the app in terms of demand, participants characteristics, practicalities and usage of MyDiaMate, and 2) to explore user experiences.

## 2 Materials and methods

The content of ‘MyDiaMate’ was developed following an iterative process, involving researchers in diabetes psychology, mental healthcare professionals specialized in diabetes, and end-users i.e. adults with diabetes (user panel). The content was largely derived from a self-help book (Snoek, 2001) and previously proven efficacious diabetes CBT-based interventions: Dia-Fit (Menting et al., 2017), HypoAware (Rondags et al., 2016a), ‘Diabetes de Baas’ (NCW et al., 2005) and ‘Diabetergestemd.nl’ (van Bastelaar et al., 2011), and was built in the eHealth Minddistrict platform (Minddistrict, 2020). The development process is described in Table 1.

**Table 1:** Development process of MyDiaMate.

(1) User input A user panel of 27 members was asked to provide input for the development of MyDiaMate (15 women; age range 45 to 64 years old; 22 type 1 diabetes, 3 type 2 diabetes). In November 2017, a meeting with 17 members of the user panel was organized to explore needs, values and wishes for MyDiaMate. Main topics discussed were: (a) *positive connotation,* information had to be positive, ‘normalizing’, e.g. “I'm not the only one dealing with emotions and struggling to keep my diabetes under control” and encouraging, e.g. “pep talk”; (b) *personalized*, information had preferably to be tailored to the needs of the individual user; (c) *goal setting*, users are able to set and monitor their goals and are encouraged to reach their goals; (d) *monitoring of blood glucose values and mood,* being able to (automatically) monitor and gain insight in blood glucose levels and mood.
(2) Content and design Between November 2017 and August 2018 the MyDiaMate project team discussed the desired structure of the app, relevant themes, and how to incorporate CBT techniques. The content of the diabetes CBT interventions Dia-Fit (Menting et al., 2017), HypoAware (Rondags et al., 2016a; Rondags et al., 2016b), ‘Diabetes de baas’ (NCW et al., 2005), ‘Elke dag diabetes’ (Snoek, 2001) and ‘Diabetergestemd.nl’ (van Bastelaar et al., 2011) was reviewed, selected and adapted to the goals and demands of the app under development.
(3) User tests In August and September 2018, the content was built in the Minddistrict platform and was tested by the MyDiaMate project team members, leading to some minor adjustments. Next, the beta version of MyDiaMate was tested by 13 members of the user panel, who filled out a questionnaire about their user experience. 4 members were invited for a *user walkthrough* session with the coordinating researcher (LM). Based on the feedback of the panel members, several technical adjustments were made before undertaking the study concerning (1) the *amount of text*, shortening of text and changes in look-and-feel, (2) *navigation and app structure,* matching the linear app structure, and *restructuring* design of the app according to flow diagram, as illustrated in Fig. 2.

### 2.1 MyDiaMate

**Fig. 1:**
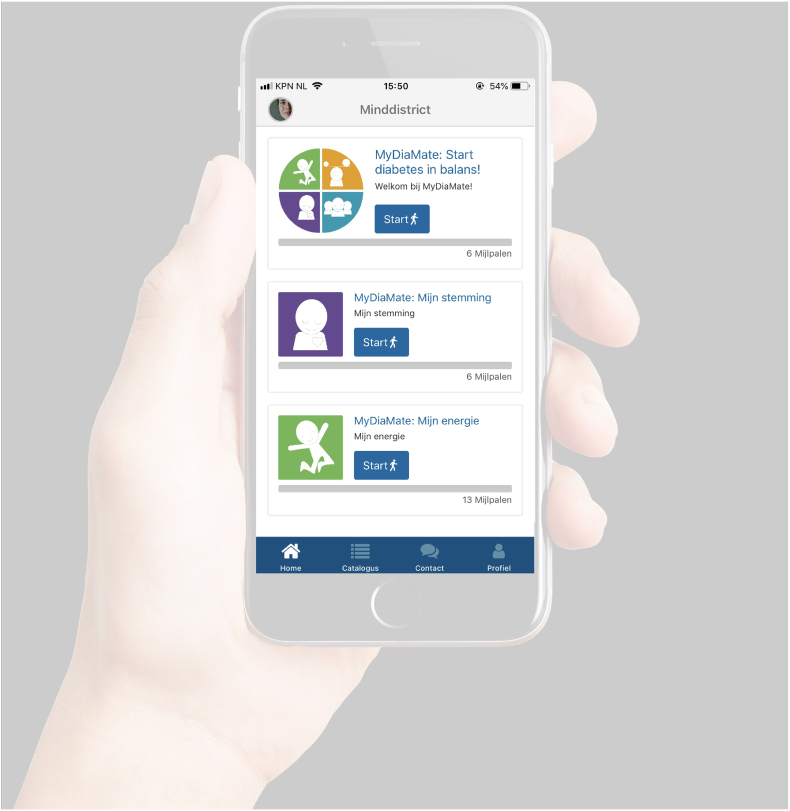
Screenshot of home page.

**Fig. 2:**
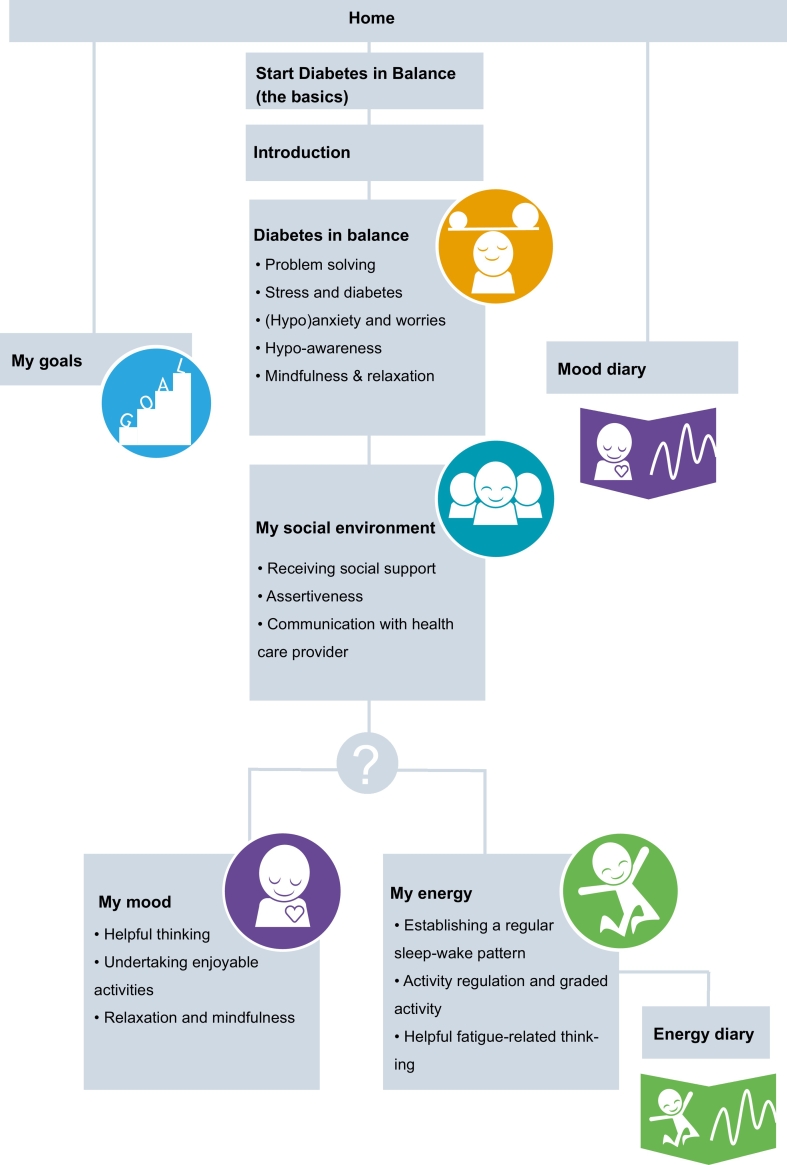
Content and structure of MyDiaMate.

Key elements of the program include psycho-education and Cognitive Behavior Therapy, offering goal setting, exercises, tips, feedback based on user-input, quotes, milestones, and diaries. The homepage of MyDiaMate is shown in Fig. 1, Fig. 2 illustrates the flow and content of MyDiaMate. MyDiaMate offers two ‘basic’ psychoeducational modules which include (1) adaptive coping and problem solving and the topics stress, (hypo)fear & worries; hypo-awareness and relaxation, called *‘Diabetes in balance’* and (2) receiving social support, communication and assertiveness, ‘*My social environment’*. This is followed by two CBT modules: (3) ‘*My mood’* including helpful thinking, undertaking enjoyable activities and relaxation and mindfulness, and (4) *‘My energy’*, including establishing a regular sleep-wake pattern, activity regulation and graded activity and helpful fatigue-related thinking. Both modules are based on evidence-based guided internet-based interventions for persons with diabetes developed by our group (van Bastelaar et al., 2011; Menting et al., 2017).

Based on preference and a short self-assessment at the end of the psychoeducational modules, users can choose to stop, or start *My mood* or *My energy*, or both with no prescribed order. The self-assessment is based on four statements, indicating either mood problems e.g. ‘*Over the last 2 weeks, ‘I had little interest or pleasure in doing things.’,* and fatigue symptoms e.g. ‘*Over the last 2 weeks, I did not do the things I would have liked to do because I was tired’*. Parallel to the CBT modules, users can enter the page *My goals* to formulate a personal goal, and ascertain to what degree they have reached their goal. Within the CBT modules, the user is encouraged to work on their goal, and use the *Mood diary* and *Energy diary* to monitor their progress. The *Energy diary* is triggered when starting with graded activity in the module *My energy*. For both diaries users receive scheduled notifications.

### 2.2 Study design

We tested the feasibility of MyDiaMate and user experiences in a non-randomized study with pre- and post-measurements, using online questionnaires and additional interviews. After online consent, participants were given free access to the app for a period of three months. Gift cards of 50 euros were randomly allotted to five participants. The Medical Ethics Committee of the VU University Medical Center approved the study protocol (2017.492).

### 2.3 Study procedure

We recruited participants from February to March 2019 by means of advertisements. Flyers were distributed among three diabetes clinics in the Amsterdam area, and we posted on social media (diabetes-related Facebook groups and Twitter) and other diabetes-related platforms, such as the website of the Dutch Diabetes Research Foundation. Participants were also recruited indirectly via the members of our user panel. People could express their interest to participate in the study by e-mail in response to which they received an information letters describing the purpose and design of the study. People were eligible if they had been diagnosed with diabetes by a physician (self-report, all types were included; diagnosed since at least 12 months), were 18 years or older, owned an Android (version 11 or higher) or iOS (version 5.0 or higher) operating smartphone, had no current diagnosis of any severe mental illness (self-report) and were interested in using the app based on the information provided in the flyer. After providing online informed consent by checking a box, participants filled out an online questionnaire at baseline, pertaining to participants' socio-demographic and clinical characteristics and psychosocial outcomes of interest. Next, they received an e-mail with instructions to log in into the Minddistrict platform and access MyDiaMate. Within the three month period data was collected on each individuals' usage of the app. Participants could decide for themselves how often and for how long they wished to use the app. At the end of the study period, participants were invited to complete online questionnaires pertaining to their psychological health, fatigue, user experience and asking for suggestions for further development. We randomly selected four participants for a telephone interview around user satisfaction that was audio recorded after consent was given.

### 2.4 Outcome measures

The primary outcomes are 1) feasibility (the number and characteristics of participants, practicalities around access, usage of the app), and 2) user experiences (user friendliness and satisfaction with the app and its features).

#### 2.4.1 Feasibility

##### 2.4.1.1 Participant characteristics

At baseline we collected sociodemographic data (age, gender, education, living status), and clinical information (type of diabetes, diabetes duration, diabetes complications, comorbidity), history or current psychological complaints, current psychological treatment. Pre- and post-three-month use of the app psychological distress and fatigue symptoms were assessed (with the 5-item ‘World Health Organization Wellbeing Index’ (WHO-5) (TRS et al., 2013), 5-item ‘Problem Areas in Diabetes 5’ (PAID-5) (McGuire et al., 2010), and the 8-item fatigue severity subscale of the ‘Checklist Individual Strength’ (CIS) (Vercoulen et al., 1999).) A WHO-5 score of less than 50 (range 0 to 100), indicates poor emotional-wellbeing (De Wit et al., 2007), a PAID score of score of 8 or higher (range 0 to 20) suggests elevated diabetes-distress (McGuire et al., 2010), and a CIS subscale score of 35 or higher (range 8 to 56) indicates fatigue severity (Goedendorp et al., 2014).

Motivation for using the app was assessed at follow-up with the question “What was the most important reason for you to start using MyDiaMate?” with answer options “(1) I wanted to improve or maintain my mental fitness; (2) I wanted to participate in research; (3) I was curious about MyDiaMate; (4) Other, namely…”.

##### 2.4.1.2 Practicalities and usage

For technical issues, participants could e-mail the research coordinator. At follow-up, participants could indicate if they encountered any technical issues regarding the app.

To determine usage of MyDiaMate, we used the reported creation and completion time-stamps of each module page to calculate the number of participants using the modules at least once, the total usage period of modules in days, and the duration in minutes of participants using the modules at least once. Time spent on a page was considered invalid when exceeding 14 min. Timestamps of the filled in diaries were used to calculate the number of participants who used the diaries at least once, the user period of diaries in days per participant, and the frequency of diary use per participant.

#### 2.4.2 User experiences

Experience and satisfaction with the app were measured using questions regarding the values attributed to the overall content, text and other features of the app. Likert-scales ranging from 1 ‘completely disagree’ to 5 ‘completely agree’ were used, with higher scores indicating more behavior change (intention) or higher satisfaction. Furthermore, participants were asked whether they would recommend the app to others with diabetes (‘no’, ‘yes’, ‘I don't know’), and were asked to grade the app on a scale from 1 to 10, with higher scores representing higher appreciation. In addition, participants were asked: “By using MyDiaMate, I came to the following conclusion: 1) I feel mentally well and I would not use MyDiaMate any further; 2) I feel mentally well, and I would like to use MyDiaMate to remain well; 3) Right now, I feel mentally well and I would use MyDiaMate again when I feel worse; 4) I need more help; 5) I don't know”. Qualitative data from the interviews and open-ended questions derived from the questionnaire were used to enrich and interpret the quantitative data.

At the end of the access period, participants were asked with open-ended question if they had any recommendations for further improvements, both in the self-reported questionnaire (all) and the interviews (*n* = 4).

With a view on the potential efficacy of MyDiaMate we documented pre-post change scores related to psychological well-being (WHO-5) (De Wit et al., 2007), diabetes-distress (PAID-5) (McGuire et al., 2010), and fatigue severity (fatigue severity subscale of the CIS) (Vercoulen et al., 1999).

### 2.5 Statistical analysis

1) Baseline measures were summarized using mean and standard deviation or frequencies and percentages in case of categorical data. Current/history of psychological complaints at baseline and total scores on the WHO-5, PAID-5, CIS fatigue severity at baseline and follow-up were calculated. Participants who completely filled out the second questionnaire after the access period were compared to those who did not by *t*-tests or Chi-square tests.

Frequency and duration of use per module and diary was summarized using the range and the median, due to the skewness of the data. Users were identified as low and high-users by median split in minutes of usage, and Chi-square analysis were used to examine if the groups differed terms of psychological measures.

2) We calculated the frequency and percentages of the user experience scores and summarized the themes of our qualitative interview findings. SPSS version 26.0 was used for all analyses.

## 3 Results

**Fig. 3:**
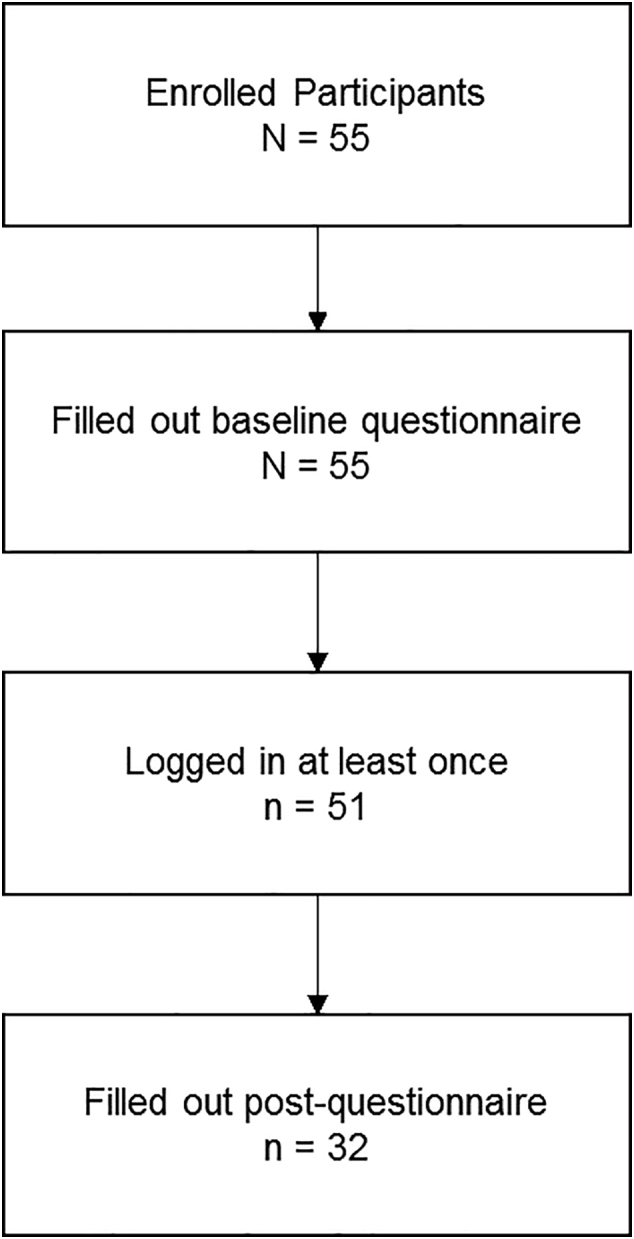
Participants flow chart.

A total of *N* = 55 eligible participants signed up for the study. Fig. 3 shows the participant flow. After the 3-month study period, *n* = 32 participants (58%) filled out the follow-up questionnaires, with no missing data within those n = 32 participants. No differences in baseline participants characteristics and psychological measures were found between those who completed the follow-up (n = 32), and those who did not (*n* = 23). The usage of the modules in minutes was significantly higher (*p* < .05) among participants who completed the follow-up questionnaire (median = 34 min, 0 to 80 min), compared to those who did not (median = 24 min, 0 to 59 min).

### 3.1 Feasibility (*N* = 55)

#### 3.1.1 Participant characteristics

Table 2 provides demographical and diabetes-related data (N = 55). Most participants were female (*n* = 39, 70.9%), higher educated (*n* = 36, 65.5%), and diagnosed with type 1 diabetes (*n* = 37, 67.3%). Mean age was 42 years, ranging from 19 to 73 years of age. Almost half of the participants indicated that they had 1 or more comorbidities (*n* = 27, 49.1%). More than 1 out of 5 were currently seeing a psychologist (*n* = 12, 21.8%). About half reported a current/history of psychological complaints and/or a low wellbeing and/or high fatigue symptoms scores.

**Table 2:** Demographical and diabetes-related characteristics of study participants (*N* = 55).

Characteristics	
Age in years, mean (range)	42 (19–73)
Female, n (%)	39 (70.9)
Educational level, n (%)	
Lower secondary education	3 (5.5)
Higher secondary education	16 (29.9)
Tertiary education (bachelor, master or equivalent)	36 (65.5)
Living status, n (%)	
Single/ divorced	12 (21
Living with parents	4 (7.3)
Partner living somewhere else	4 (7.3)
Living with partner/ married	35 (63.6)
Type of diabetes, n (%)	
Type 1	37 (67.3)
Type 2	9 (16.4)
Other (Mody or LADA)	9 (16.3)
Diabetes duration in years, mean (SD), median (range)	15.7 (13.1), 13.0 (0–51)
Diabetes complications, n (%)	
None	42.7 (15.6), 46 (19–73)
1 or more	10 (18.2)
Comorbidity, n (%)	
None	28 (50.9)
1	20 (36.4)
2 or more	7 (12.7)
History or current psychological complaints, (n%)	27 (49.1)
History of psychological diagnosis, n (%)	
Anxiety	3 (5.5)
Depression	12 (21.8)
Chronic fatigue	3 (5.5)
Burn-out	5 (9.1)
Other	7 (12.7)
Don't know	4 (7.3)
Current psychological help, n (%)	
From psychologist	12 (21.8)
Current use psychiatric medication, n (%)	4 (7.4)
Elevated scores of baseline questionnaires, n (%)	
WHO 5 (≤50)	25 (45.5)
PAID 5 (≥8)	29 (52.7)
CIS fatigue severity (≥35)	35 (63.6)

#### 3.1.2 Practicalities and usage

No major issues were encountered with respect to the technology or download/onboarding procedure. Some minor issues were reported: slow start (n = 2), audio stopped, when screen went on standby (n = 1).

Within the period of three months, *n* = 51 (92.7%) opened one or more modules of MyDiaMate, and used the modules 1 to 80 min (median = 28 min) in a period of 1 to 92 days (median = 10 days). Data of usage of participants who logged in at least once (n = 51) is displayed in Table 3. Of those participant, almost all (*n* = 50, 98.0%) opened the basic module *Diabetes in balance*, and 32 (62.7%) and arrived at the final page of this module. The CBT modules ‘*My mood’* and ‘*My energy’* were opened by *n* = 40 (78.4%) and 32 (62.7%) participants, respectively, and completed by *n* = 21 (52.5%) and 9 (28.1%) of the participants. ‘My goals’ was accessed by *n* = 27 (52.9%) participants. Most goals were related to healthy life style behaviors: increasing exercise (*n* = 14) and healthy eating (*n* = 8). The ‘Mood diary’ was filled in once to 156 times (median = 5 times) by *n* = 48 participants, and the ‘Energy diary’ was filled in twice up to 16 times (median = 6 times) by *n* = 8 participants who entered the module ‘My energy’. The low and high-user groups did not differ in terms of their psychological profile.

**Table 3:** Usage data (n = 51).

User engagement		
Number of participants using the modules at least once, n (%)	Opened	Closed
Introduction	50 (98.0)	49 (96.1)
Diabetes in balance	46 (90.2)	33 (64.7)
My social environment	32 (62.7)	32 (62.7)
My mood	40 (78.4)	21 (41.2)
My energy	32 (62.7)	9 (17.6)
My goals	27 (52.9)	n.a.
Total usage period of modules in days, median (range), n = 51	10.0 (1.0–91.7)	
Duration in minutes of participants using the modules at least once, median (range)		
Introduction (4 pages)	3.2 (0.3–14.8)	
Diabetes in balance (17 pages)	10.3 (1.0–36.6)	
My social environment (3 pages)	4.0 (0.8–20.9)	
My mood (28 pages)	11.5 (0.3–35.6)	
My energy (38 pages)	11.1 (0.1–27.4)	
Total	28.4 (1.0–80.1)	
Number of participants who used diaries at least once, n (%)		
Mood diary	48 (94.1)	
Energy diary	8 (25% of the 32 participants who opened My energy)	
User period of diaries in days per participant, median (range)		
Mood diary	39 (1–93)	
Energy diary	23 (1–52)	
Frequency of diary use per participant, median (range)		
Mood diary	18 (1–156)	
Energy diary	8 (2–16)	

### 3.2 User experiences (*n* = 32)

Data on the user experience of MyDiaMate gathered at follow-up (n = 32) are summarized in Table 4.

**Table 4:** Usability (user behavior, experience and satisfaction) rated by participants (n = 32).

Theme	Question			
		(Totally) agree	Neutral	(Totally) disagree
Learnability	I learned something new by using MyDiaMate, n (%)	13 (40.7)	8 (25.0)	11 (34.4)
Self-efficacy	MyDiaMate gives me the confidence to work on my mental fitness, n (%)	12 (37.5)	10 (31.3)	10 (31.3)
Motivation	By using MyDiaMate, I feel supported in preserving or improving my mental fitness, n (%)	8 (25.0)	16 (50.0)	8 (25.0)
	MyDiaMate stimulates me to think about how I can preserve or improve my mental fitness, n (%)	18 (56.3)	7 (21.9)	7 (21.9)
	MyDiaMate encourages me to take actions which might be beneficial to my mental fitness, n (%)	14 (43.7)	6 (18.8)	12 (37.5)
Overall	**I find MyDiaMate…, n (%)**			
	Trustworthy	20 (62.5)	10 (31.3)	2 (6.3)
	Informative	24 (75.0)	4 (12.5)	4 (12.5)
	Useful	12 (37.5)	7 (21.9)	13 (40.6)
	Clear	19 (59.4)	8 (25.0)	5 (15.6)
	Attractive	8 (24.0)	13 (40.6)	11 (34.4)
	Easy to use	17 (53.1)	8 (25.0)	7 (21.9)
Text	**I find the text of MyDiaMate…, n(%)**			
	Appealing	18 (56.3)	8 (25.0)	6 (18.8)
	Positive	20 (62.5)	9 (28.1)	3 (9.4)
	Put things in perspective	15 (46.9)	14 (43.8)	3 (9.4)
	Negative	0 (0.0)	7 (21.9)	25 (78.1)
	Confusing	3 (9.4)	8 (25.0)	21 (65.6)
	Pedantic	7 (21.9)	8 (25.0)	17 (53.1)
	Recognizable	21 (65.6)	8 (25.0)	3 (9.4)
	Confrontational	6 (18.8)	15 (46.9)	11 (34.4)
	Complicated	1 (3.1)	6 (18.8)	25 (78.1)
	Length^a^	2 (6.3)	17 (53.1)	13 (40.6)
Illustrations	**I find the illustrations of MyDiaMate…, n(%)**			
	Appealing	10 (31.3)	18 (56.3)	4 (12.5)
	Quantity^a^	9 (28.1)	19 (59.4)	4 (12.5)
Exercises	I understand how I can execute the exercises, n(%)^b^	20 (62.5)	2 (6.3)	1 (3.1)
	I find the exercises useful, n(%)^b^	10 (31.3)	10 (31.3)	3 (9.4)
Dairies	I understand how I can use the diaries, n(%)^c^	19 (59.4)	2 (6.3)	4 (12.5)
	The notifications to fill out my diary help me to keep track my mood or energy.	9 (28.1)	6 (18.8)	12 (37.5)
Recommendation	**I would recommend MyDiaMate to others with diabetes, n (%)**			
	No	9 (28.1)		
	Yes	13 (40.6)		
	I don't know	10 (31.3)		
Grade	On a scale from 1 to 10, I would give MyDiaMate the grade, mean (SD), range	6.2 (1.9), 2–10		
Conclusion	**By using MyDiaMate, I came to the following conclusion, mean (SD)**			
	I feel mentally well and I would not use MyDiaMate any further.	14 (43.8)		
	I feel mentally well, and I would still use MyDiaMate to stay this way.	6 (18.8)		
	Right now, I feel mentally well and I would use MyDiaMate again when I feel worse.	2 (6.3)		
	I need more help.	5 (15.6)		
	I don't know.	4 (12.5)		
	Missing	1 (3.1)		

a Scale: ‘too little’; ‘exactly right’; ‘too much’.

b Descriptives of the 23 participants who executed at least one exercise.

c Descriptives of the 25 participants who used a diary at least once.

Preserving or improving mental fitness (*n* = 13, 40.6%) was the primary reason for using MyDiaMate, followed by wanting to participate in research (*n* = 11, 34.4%), and curiosity (*n* = 8, 25.0%). Most participants found the app informative (*n* = 24, 75.0%), trustworthy (*n* = 20, 62.5%), and clear (*n* = 19, 59.4%). Twenty-five percent felt supported in preserving or improving their mental fitness (n = 8), about half of the participants was neutral. MyDiaMate stimulated most participants to think about how they can preserve or improve their mental fitness (*n* = 18, 56.3%), e.g. “gives insight”.

Less than half of the participants did not find MyDiaMate useful (*n* = 13, 40.6%). For example, one participant noted: “*the app is not useful for me, because I do not want to be occupied with my diabetes the whole day*”. Those who did find the app of use, mentioned the app was “helpful” and “easily accessible and effective”. 40.6% would recommend the app to other persons living with diabetes. Overall, participants rated the app on average a with a 6.2 (range 2 to 10) on a 1–10 scale.

Suggestions for further development of the app were put forward by 19 participants. Five participants would like to have access to more information, of which three suggested the possibility to use drop-down menus or links. One participant suggested videos to clarify or reduce text. Four participants found it unclear how and when to use the app, or specifically where to find information. Six participants found the notifications unclear, not helpful or even disruptive. One participant would like to have the option to receive feedback on the diaries. Other suggestions were the possibility to link the mood diary to a blood glucose monitor (*n* = 1), and having the option to communicate with other persons with diabetes via a chat feature (n = 1).

Table 5 shows the observed pre-post scores of psychological outcomes of interest of the 32 participants that completed the study.

**Table 5:** Change scores of psychological outcomes, *n* = 32.

		Mean score (SD)	
Construct	Measure	Pre	Post
Well-being	WHO 5	54.0 (20.1)	57.9 (20.9)
Diabetes distress	PAID 5	6.7 (4.2)	6.0 (3.6)
Fatigue	CIS fatigue severity	36.2 (11.8)	32.6 (13.4)

WHO, World Health Organization Wellbeing Index; PAID, Problem Areas in Diabetes.

CIS, Checklist Individual Strength.

## 4 Discussion

We developed the web-based self-help MyDiaMate for adults with diabetes and conducted a feasibility study looking at uptake, patient characteristics, practicalities, usage and user experiences. The number of patients that signed up for the study (*n* = 55) within a recruitment period of 2 months seems to confirm a demand for this type of self-help. Future research should further explore the potential reach and adoption of MyDiaMate in a large scale study.

We found evidence to support feasibility and overall positive user experiences of MyDiaMate. No major technical or practical obstacles were encountered in the process of accessing, onboarding or using the app as intended. The findings with regard to the user experiences are encouraging. Overall, participants experienced MyDiaMate as informative, trustworthy and clear. The text was generally appreciated as appealing, positive and recognizable. However, slightly less than half of the participants found that there was too much text to read. The app provides mostly written information and may require (too) much cognitive effort, at least for some. Limiting and replacing text by providing videos or animations may be helpful in this respect and experienced as more engaging (Cheung et al., 2017; Stanczyk et al., 2014). The way the app is structured, starting with a basic module followed by two optional modules provides clarity on the route to follow. This probably partly explains that most participants experienced the app as ‘easy to use’, but such a fixed structure may not be preferred by everyone. For example, users who look for certain information may prefer to go directly to the content within a few clicks, without having to go through the large parts of the program. As a result, users may not be able to directly access the sought information on psychosocial issues and therefore feel less supported. Offering more flexibility would appear particularly salient for the psychoeducational modules of the app.

The app seems to stimulate most users to think about how to improve and preserve their mental fitness in the context of living with and having to self-manage diabetes. However, not everyone reported to have felt supported, which may be related to the fact that the app does not connect to a professional or support network. Incorporating the option of a group chat, may prove helpful to some to feel more connected and emotionally supported (Brouwer et al., 2011).

The app offers prescheduled notifications, reminding the user to fill out the mood and/or energy diary. Such push notifications can increase app engagement, but only few in our study found them to be helpful. Tailoring push notifications to the individual's needs and preferences may help to increase acceptance and effectiveness (Bidargaddi et al., 2018).

### 4.1 Strengths and limitations

We involved multiple professionals and patients in developing MyDiaMate. Another strength worth mentioning is the fact that much of the content is evidence-based. As noted, the app was offered within a research context, which might have resulted in a selection bias in participants (e.g. ethnicity and educational level), limiting the generalizability of our findings.

Based on our results we cannot yet define for whom the app is suited and for whom it is not.

Participants were all self-referred and attracted predominantly higher educated females with type 1 diabetes, with about half reporting to have psychological difficulties. This finding is in line with previous psychological intervention studies in diabetes (van Bastelaar et al., 2011; Menting et al., 2017; Rondags et al., 2016a; NCW et al., 2005; Liddon et al., 2018).

The over representation of type 1 diabetes patients is not unexpected, given the fact that overall they are younger and many are a member of an online type 1 diabetes community, For this study we advertised through social media and handed out flyers in outpatient clinics where the majority of patients has type 1 diabetes.

Over 40% joined the study with the aim to preserve and improve their mental health and energy, in line with the purpose of MyDiaMate. At this stage, based on the observed pre-post change scores in our small sample we cannot draw conclusions regarding the efficacy nor of any potential harm of MyDiaMate. For further research it would be helpful to determine a minimum level of engagement, expressed in time or completed exercises, to experience improvement in mental fitness and energy level.

## 5 Conclusion

This study showed promising results with respect to feasibility and user experiences of the unguided self-help app ‘MyDiaMate’, designed to help persons with diabetes reduce and possibly prevent psychological distress and fatigue. Future research should aim to examine its effectiveness and to explore ways to further personalize content and features based on individual preferences and needs.

## Funding

We are grateful for the funding received form the 10.13039/501100003092Dutch Diabetes Research Foundation (project number: 2016.30.011).

## Declaration of competing interest

The authors declare that they have no conflict of interest.
